# Electrochemically deposited gallium oxide nanostructures on silicon substrates

**DOI:** 10.1186/1556-276X-9-120

**Published:** 2014-03-17

**Authors:** Norizzawati Mohd Ghazali, Mohamad Rusop Mahmood, Kanji Yasui, Abdul Manaf Hashim

**Affiliations:** 1 Malaysia-Japan International Institute of Technology, Universiti Teknologi Malaysia, Jalan Semarak, Kuala Lumpur 54100, Malaysia; 2 Faculty of Electrical Engineering, Universiti Teknologi MARA, Shah Alam, Selangor 40450, Malaysia; 3 Department of Electrical Engineering, Nagaoka University of Technology, Kamitomioka-machi, Nagaoka, Niigata 940-2137, Japan; 4 MIMOS Berhad, Technology Park Malaysia, Kuala Lumpur 57000, Malaysia

**Keywords:** Electrochemical deposition, Gallium oxide, Liquid phase, Nanorod, Gallium nitride

## Abstract

We report a synthesis of β-Ga_2_O_3_ nanostructures on Si substrate by electrochemical deposition using a mixture of Ga_2_O_3_, HCl, NH_4_OH, and H_2_O. The presence of Ga^3+^ ions contributed to the deposition of Ga_2_O_3_ nanostructures on the Si surface with the assistance of applied potentials. The morphologies of the grown structures strongly depended on the molarity of Ga_2_O_3_ and pH level of electrolyte. β-Ga_2_O_3_ nanodot-like structures were grown on Si substrate at a condition with low molarity of Ga_2_O_3_. However, Ga_2_O_3_ nanodot structures covered with nanorods on top of their surfaces were obtained at higher molarity, and the densities of nanorods seem to increase with the decrease of pH level. High concentration of Ga^3+^ and OH^-^ ions may promote the reaction of each other to produce Ga_2_O_3_ nanorods in the electrolyte. Such similar nature of Ga_2_O_3_ nanorods was also obtained by using hydrothermal process. The grown structures seem to be interesting for application in electronic and optoelectronic devices as well as to be used as a seed structure for subsequent chemical synthesis of GaN by thermal transformation method.

## Background

Since the last two decades, due to its unique properties, gallium oxide (Ga_2_O_3_) has been considered as a candidate to be used in electronic and optoelectronic devices [1]. Ga_2_O_3_ have been classified into five different polytypes, which are denoted as alpha (α), beta (β), gamma (γ), delta (δ), and sigma (ϵ) [2,3]. Among all of these polytypes, β-Ga_2_O_3_ is stable and α-Ga_2_O_3_ is basically meta-stable [4]. β-Ga_2_O_3_ behaves as an insulator at room temperature, while it shows semiconducting properties at temperature above 500°C [5]. Generally, the as-deposited β-Ga_2_O_3_ layer shows amorphous structures, but depending on the deposition method, the crystallization of the grown film could be achieved by annealing at high temperature above 700°C [5].

β-Ga_2_O_3_ possesses wide band gap of 4.8 eV and high breakdown field of 8 MVcm^-1^ which makes it possible to be fabricated for power devices such as metal-semiconductor field-effect transistor (MESFETs) [2,6]. The characteristic of β-Ga_2_O_3_ that is chemically and thermally stable also makes it as a good candidate for sensing application under a harsh environment [7,8]. In recent years, Ga_2_O_3_ has been studied to be used as the seed material for chemical synthesis of gallium nitride (GaN) by thermal transformation method [9].

Ga_2_O_3_ has been successfully grown on various kinds of substrates such as sapphire and silicon (Si) substrate [3,10]. However, sapphire substrate is expensive and is not available in large wafer size [11]. Si substrate is considered to be more preferable due to the availability of large wafer size and low price [12]. In addition, the hybrid integration of semiconductor-based devices on Si platform seems to be very attractive towards the so-called More-than-Moore technology [13].

Up to date, several vapor phase techniques such as chemical vapor deposition (CVD), metal organic chemical vapor deposition (MOCVD), and molecular beam epitaxy (MBE) have been widely used to synthesize β-Ga_2_O_3_ thin films and nanostructures [10,14-16]. Only few works report the synthesis Ga_2_O_3_ by liquid phase techniques such as hydrothermal and electrochemical techniques [17-20]. Such liquid-phase techniques seem to provide several advantages not only the high controllability of thicknesses and morphologies of the grown structures due to less number of growth parameters but also low-temperature and low-cost growth techniques [21].

In this paper, we report the growth of β-Ga_2_O_3_ nanostructures using a mixture of gallium oxide (Ga_2_O_3_), hydrochloric acid (HCl), ammonia water (NH_4_OH), and deionized (DI) water by simple electrochemical deposition process. The effects of Ga_2_O_3_ molarity and pH of a mixture on the morphological, structural, and optical properties were studied.

## Methods

A mixture of Ga_2_O_3_ (99.99%), HCl (36%), NH_4_OH (25%), and DI water was used as an electrolyte. Since Ga_2_O_3_ is insoluble in water, HCl is added to dissolve Ga_2_O_3_. The preparation of electrolyte was done as follows. First, Ga_2_O_3_ was dissolved in 1.5 ml HCl. Then, 6.5 ml DI water was added into the solution, followed by NH_4_OH as a precipitator, so that the pH of the mixture could be easily adjusted. The growth was done in electrolyte with different pH values of 4, 6, 8, and 10. The molarity of Ga_2_O_3_ was varied from 0.05 to 1.0 M. The deposition was done at a constant current density of 0.15 A/cm^2^. Si (100) with resistivity of 15 to 25 Ω · cm was used as the substrate. The substrate was cleaned with modified RCA cleaning using ethanol, acetone, and DI water prior to the deposition in order to remove a native oxide layer. As a comparison, a hydrothermal growth in non-pressurized container using the same composition of electrolyte with pH of 8 and Ga_2_O_3_ molarity of 0.05 M at temperature of 80°C was also carried out. The growth time was fixed at 2 h.

**Figure 1:**
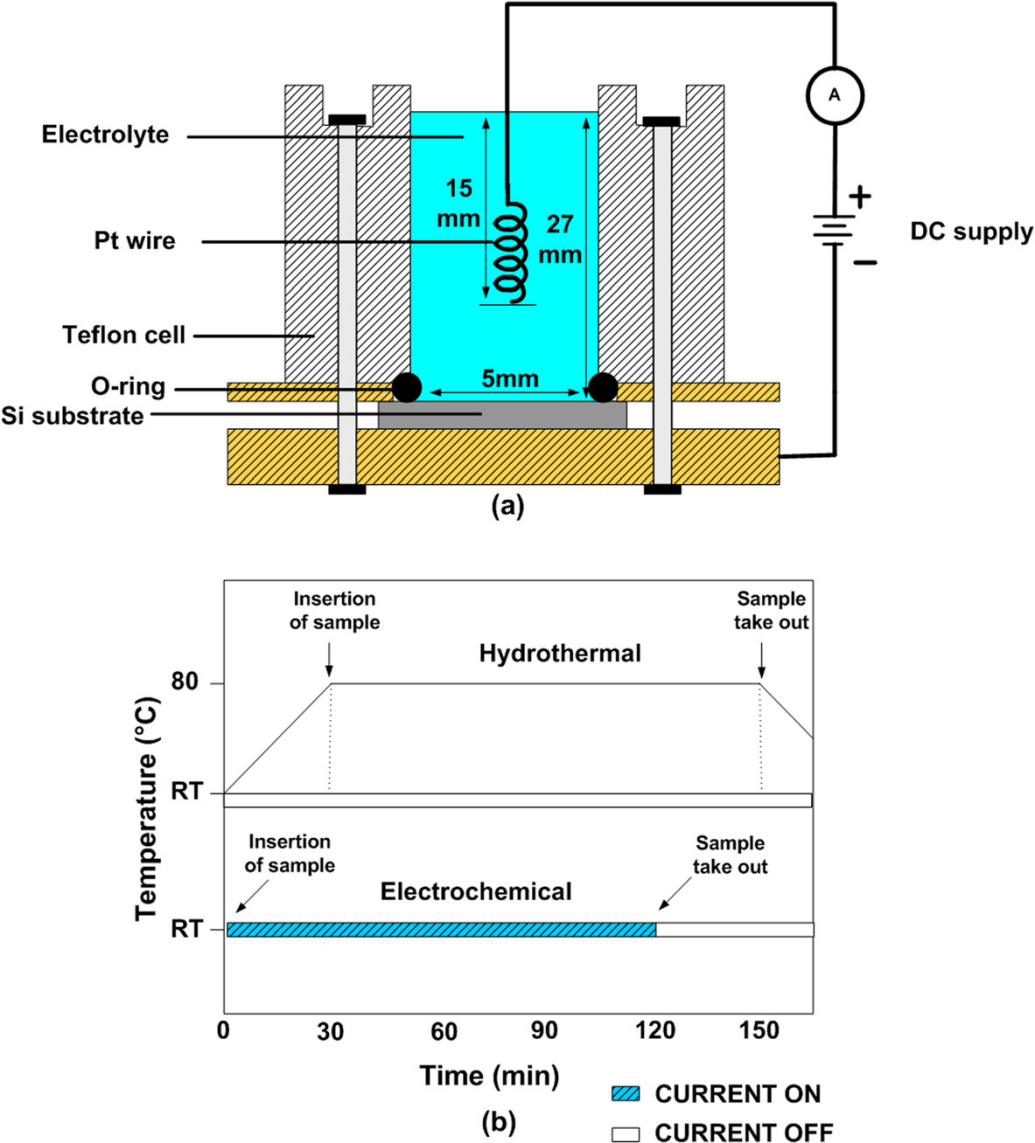
Schematic of (a) electrochemical setup and (b) growth time chart.

Figure 1a,b shows the schematic of experimental setup of two-terminal electrochemical process and the growth time chart, respectively. In this electrochemical process, a platinum (Pt) wire was used as an anode and Si substrate as a cathode. After the deposition, the sample was dipped into the DI water to remove any unwanted residue.

The grown structures was characterized using scanning electron microscope (SEM, Hitachi SU8083, Chiyoda-ku, Japan), energy dispersive X-ray spectroscopy (EDX), X-ray diffractometer (XRD, Bruker, AXES, D8 Advance, Bruker Cooperation, Billerica, MA, USA) Fourier transform infrared spectroscopy (FTIR, Agilent 660, Santa Clara, CA, USA), and photoluminescence spectroscopy (PL, Horiba Jobin Yvon, Kyoto, Japan, excitation at 325 nm with He-Cd laser source).

## Results and discussion

**Figure 2:**
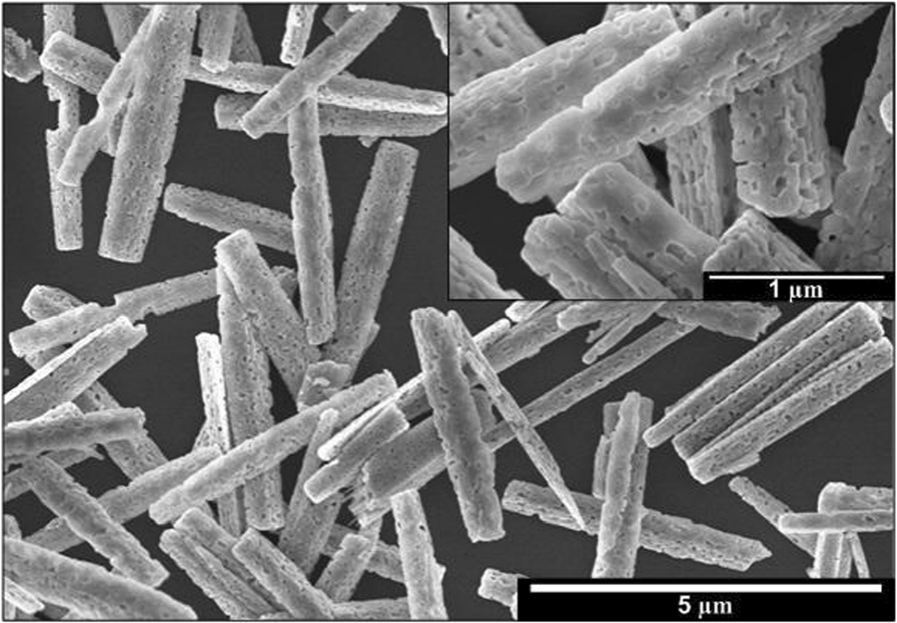
SEM images of hydrothermally grown structure.

Figure 2 shows the SEM images of the grown nanostructures on Si by hydrothermal process. The length of grown Ga_2_O_3_ nanorods was estimated to be in the range of 3,500 to 4,000 nm, and the diameters were in the range of 200 to 700 nm. These values are summarized in Table 1. SEM images clearly show that the formed nanorods were just laid down on the Si surface. This indicates that the growth of nanorods occurs in the heated electrolyte, and the growth has not been able to be induced or aligned on the Si substrate in non-pressurized container. The EDX spectra (data not shown) show that the grown nanorods contain Ga and O elements, indicating the possible formation of Ga_2_O_3_ nanorods. Temperature in hydrothermal process plays an important role in controlling the size and the length of the nanostructures, as also reported by Pei et al*.*[17]. They observed the decrease in diameter and length of the rods with the decrease of temperature. In this case, the fluctuation of the nanorods size as observed in Figure 2 is possibly due to the pre-heating process, i.e., before reaching the heating temperature of 80°C. During the pre-heating process, small quantity of nanorods start to form and the quantity increases with the temperature and time. That is the main reason for the fluctuation of nanorod sizes.

**Table 1:** **Shape**, **diameter, and length of hydrothermally and electrochemically grown structures**

**Technique**	**Ga2O3**	**pH**	**Shape**	**Diameter**	**Length**
Hydrothermal	0.05 M	8	Nanorods	200 to 700 nm	3,500 to 4,000 nm
	0.05 M	4	Nanodots	100 to 700 nm	-
		6		50 to 700 nm	-
		8		40 to 700 nm	-
		10		40 to 700 nm	-
Electrochemical	0.1 M	4	Nanodots	200 to 2,000 nm	-
		6		200 to 1,800 nm	-
		8		100 to 1,700 nm	-
		10		50 to 500 nm	-
	0.5 M	4	Nanorods (high density)	-	-
			Unable to determine nanorod size		
		6	Unable to determine nanorod size	-	-
		8	Nanodots	100 to 1,300 nm	-
			Nanorods (low density)	150 to 400 nm	1,500 to 3,000 nm
		10	Nanodots	50 to 700 nm	-
	1.0 M	4	Nanorods (high density)	200 to 500 nm	2,000 to 4,500 nm
		6	Nanodots	200 to 1,000 nm	-
			Nanorods (medium density)	200 to 800 nm	1,000 to 4,200 nm
		8	Nanodots	200 to 1,000 nm	-
			Nanorods (medium density)	200 to 500 nm	1,000 to 3,900 nm
		10	Nanodots	200 to 1,000 nm	-
			Nanorods (low density)	200 to 800 nm	1,000 to 3,700 nm

The possible chemical reactions that take place in the electrolyte by hydrothermal process can be represented as the following:

(1) $${\mathrm{Ga}}_{2}O_{3}+6\mathrm{HCl}\to 2{\mathrm{GaCl}}_{3}+3H_{2}O$$

(2) $${\mathrm{GaCl}}_{3}+3{\mathrm{OH}}^{-}\to \mathrm{Ga}{\left(\mathrm{OH}\right)}_{3}+3{\mathrm{Cl}}^{-}$$

(3) $$\mathrm{Ga}{\left(\mathrm{OH}\right)}_{3}\to \mathrm{GaO}\left(\mathrm{OH}\right)+H_{2}O$$

(4) $$2\mathrm{GaO}\left(\mathrm{OH}\right)\to {\mathrm{Ga}}_{2}O_{3}+H_{2}O$$

As shown in Equation 1, GaCl_3_ compound is produced from the mixture of Ga_2_O_3_ and HCl. With the addition of NH_4_OH into a mixture of Ga_2_O_3_ and HCl, Ga(OH)_3_ precipitates to form supersaturated solution as represented in Equation 2 [22]. Then, the solution achieves the equilibrium state as shown in Equation 3 [22]. The saturation of the solution increases gradually when the pH value is increased to form Ga_2_O_3_ in the solution as shown in Equation 4 [22].

Usually, the hydrolysis process of metal cations in aqueous solution and their kinetics are influenced by temperature. Water acts effectively as a reactant in hydrolysis process at higher temperature. At higher temperature, OH^-^ ions are sufficiently generated in water and Ga_2_O_3_ can be easily synthesized. When heat is applied under hydrolysis process, Ga^3+^ ions are separated because of the decrease of viscosity and the increase of ionic product of water. The solubility of solutes changes when heat is applied that makes chemical reaction occur in the solutions [19]. When the solution is in supersaturation condition as shown in Equation 4, the solid particles are formed resulting to the crystal growth of Ga_2_O_3_ in the electrolyte.

In electrochemical deposition process, similar growth process as hydrothermal process takes place in aqueous gallium oxide solution. The possible reactions that occur in anode and cathode are summarized in the following equations:

(5) $$\mathrm{Cathode}:4{\mathrm{Cl}}^{-}+4H_{2}O\to 4\mathrm{HCl}+4{\mathrm{OH}}^{-}$$

(6) $$2{\mathrm{Ga}}^{3+}+4{\mathrm{OH}}^{-}+2e\to {\mathrm{Ga}}_{2}O_{3}+H_{2}O+H_{2}$$

(7) $$\mathrm{Anode}:H_{2}O\to 1/2O_{2}+2H^{+}+2e$$

When current is applied, a mixture of Ga_2_O_3_, HCl, NH_4_OH, and H_2_O that was dissociated into ions was transported in the electrolyte. OH^-^ ions are produced near the cathode electrode in aqueous gallium oxide solution when the voltage is applied as shown in Equation 5. Then, the Ga^3+^ ion reacts with OH^-^ ions to generate Ga_2_O_3_ nanostructure on Si substrate as represented in Equation 6. The presence of high concentration of Ga^3+^ ions contributes to the effective and uniform deposition on the silicon surface.

**Figure 3:**
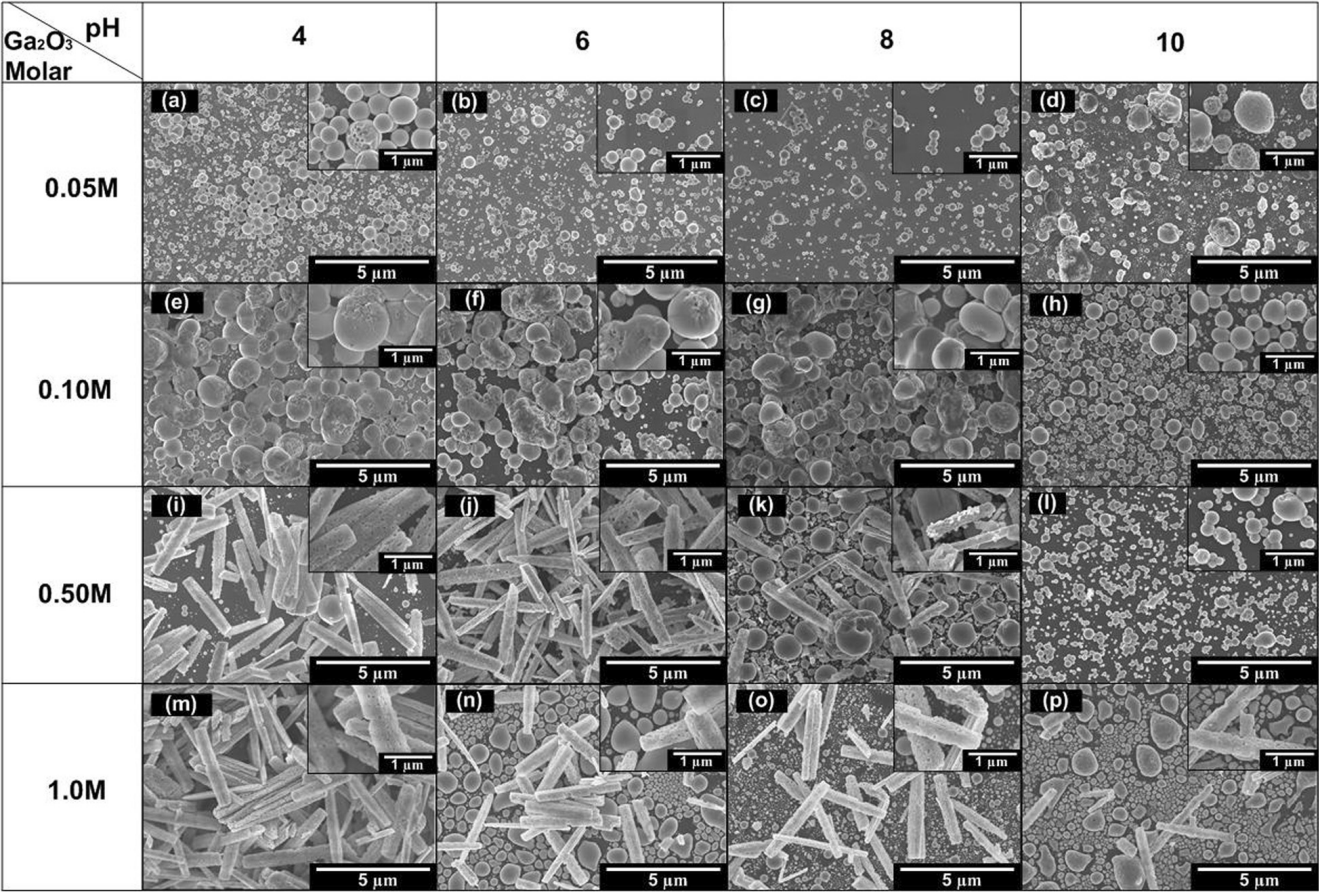
**SEM images of electrochemically grown structures with different molarities of Ga**_**2**_**O**_**3 **_**and pH levels of electrolyte.** Inset is an enlarged view.The respective molarity and pH level for a,b,c,d,e,f,g,h,i,j,k,l,m,n,o,p are indicated in the left and top of figure

Figure 3a,b,c,d,e,f,g,h,i,j,k,l,m,n,o,p shows the SEM images of the grown structures on Si by electrochemical process at room temperature with different molarities of Ga_2_O_3_ and pH values. At a low molarity of 0.05 and 0.1 M as shown in Figure 3a,b,c,d,e,f,g,h, Ga_2_O_3_ nanodot-like structures were deposited on Si substrate in both acidic and alkaline conditions. A high density Ga_2_O_3_ nanodot-like structure was deposited on Si substrate at 0.1 M, compared to that at 0.05 M, and the diameter of the deposited Ga_2_O_3_ nanodot structure in acidic condition is slightly larger compared to nanodot structure in alkaline condition. These results are similar to the results reported by Zhang et al. where they claimed that nanostructures deposited in solution with low pH value generated larger size compared to nanostructures deposited in solution with high pH value [23]. The diameters of the grown nanodot structures are summarized in Table 1.

Interestingly, when the molarity of Ga_2_O_3_ was increased to 0.5 and 1.0 M, Ga_2_O_3_ nanorods started to form in the electrolyte and these nanorods were found to lay down on the top surface of substrate grown with nanodot structures as shown in Figure 3i,j,k,l,m,n,o,p. The density of Ga_2_O_3_ nanorods significantly increase with the decrease of pH level for both Ga_2_O_3_ molarities of 0.5 and 1.0 M. It is noted here that at high pH level, surface with Ga_2_O_3_ nanodot structure without or with less density of nanorods was obtained for both molarities of Ga_2_O_3_. It can be speculated that since the concentrations of Ga^3+^ and OH^-^ ions are extremely high in the acidic electrolyte, they may easily react with each other to produce Ga_2_O_3_ nanorods. The diameter and length of the formed nanorods are also summarized in Table 1.

**Figure 4:**
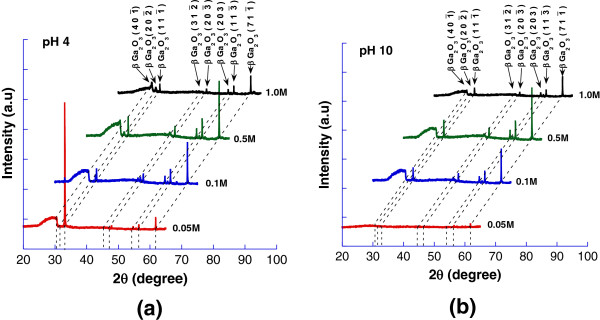
**XRD spectra electrochemically grown structures.** At **(a)** pH 4 with various molarities of Ga_2_O_3_ and **(b)** pH 10 with various molarities of Ga_2_O_3_.

Figure 4a,b shows the XRD spectra of the grown Ga_2_O_3_ nanostructures as a function of Ga_2_O_3_ molarity at pH 4 and pH 10, respectively. Five prominent peaks which correspond to β-Ga_2_O_3_$\left(11\bar{1}\right)$, β-Ga_2_O_3_$\left(20\bar{3}\right)$, β-Ga_2_O_3_ (203), β-Ga_2_O_3_$\left(11\bar{3}\right)$, and β-Ga_2_O_3_$\left(71\bar{1}\right)$ were observed at 33.4°, 47.1°, 54.5°, 56.4°, and 61.2°, respectively, in all samples grown with molarity of 0.1 to 1.0 M for both low and high pH level (JCPDS 41-1103). From these results, the growth of β-Ga_2_O_3_ was confirmed.

**Figure 5:**
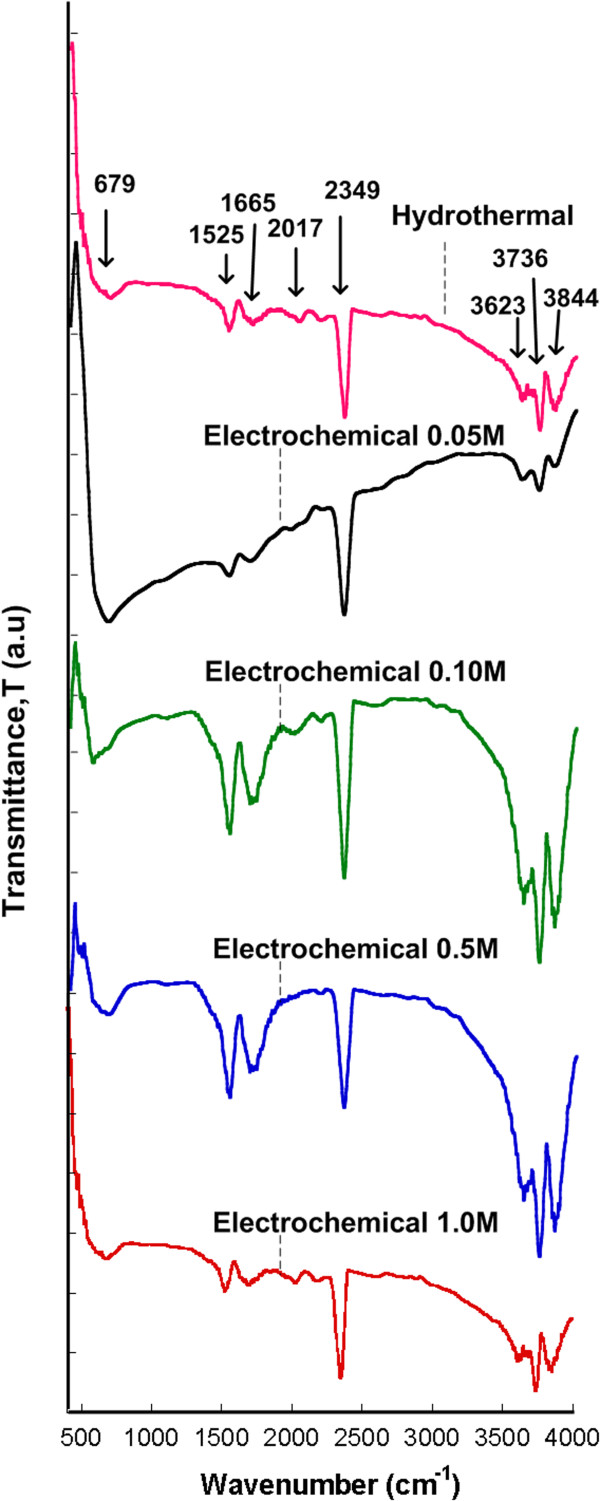
**FTIR spectra of hydrothermally and electrochemically grown structures.** FTIR spectra of hydrothermally (pH = 8, T = 80°C, molarity of Ga_2_O_3_ = 0.05 M, time = 2 h) and electrochemically grown structures (pH = 8, *J* = 0.15 A/cm^2^, molarity of Ga_2_O_3_ = 0.05 to 1.0 M, time = 2 h).

Figure 5 shows the FTIR spectra measured in the range of 400 to 4,000 cm^-1^ for samples grown by both hydrothermal and electrochemical techniques. The band with valley peak at 679 cm^-1^ was observed for all samples which can be assigned to Ga_2_O_3_ (Ga-O bonding) [20]. The bands with valley peaks at 1,525, 1,665, 2,027, and 2,349 cm^-1^ are considered to have resulted from the absorbed atmospheric CO_2_, which occurs during the preparation and processing of FTIR samples in the ambient atmosphere [24]. The bands at 3,623, 3,736, and 3,844 cm^-1^ are assigned to the H-O-H stretching and O-H stretching which are formed during the deposition in an aqueous solution [25]. The measured transmittance further confirmed the deposition of Ga_2_O_3_ structure on Si substrate.

**Figure 6:**
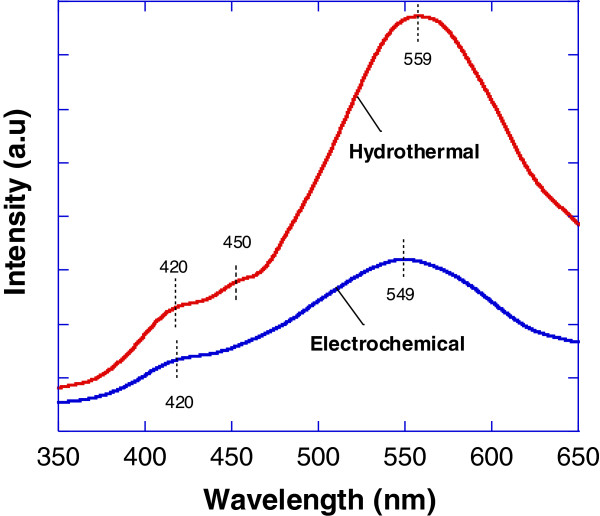
RT PL spectra of hydrothermally and electrochemically grown structures.

Figure 6 shows the measured room temperature (RT) PL spectra of β-Ga_2_O_3_ deposited by hydrothermal (pH = 8, molarity of Ga_2_O_3_ = 0.05 M, T = 80°C) and electrochemical (pH = 8, molarity of Ga_2_O_3_ = 0.5 M) methods. For hydrothermally grown sample, two shoulder's peaks at 420 (2.95 eV) and 450 nm (2.76 eV) which correspond to blue emission as also reported by Villora et al*.*[26] were observed. Villora et al*.* claimed that this blue emission was attributed to the recombination of an electron from a donor formed by oxygen vacancies and a hole of an acceptor formed by either gallium vacancies or gallium-oxygen vacancy pairs [23,27]. Similarly, for electrochemically grown sample, a shoulder at approximately 420 nm was also observed. The peaks at 559 nm (2.21 eV) and 549 nm (2.26 eV) for hydrothermal and electrochemical samples, respectively, which correspond to green emission was also clearly observed. These peaks were also similar to the reported results by Zhang et al. [28]. These peaks seem to be at red-shifted position about 50 to 60 nm compared to the reported peak of 500 nm (2.48 eV) by Villora et al*.*[26]. Binet and Gourier also reported similar red-shifting of 30 to 40 nm [29]. This green emission was proposed to be associated with self-trapped or bound excitons by Villora et al. [26].

The chemical reactions by both methods have been described in the previous section. The formation of Ga_2_O_3_ was confirmed by using EDX, XRD, FTIR, and PL measurement. From the EDX spectra, only Ga and O element were detected. There is no Cl element observed in the spectra even though HCl was added during the preparation of the electrolyte. XRD results also show that the grown structure is only Ga_2_O_3_ compound. There is no gallium chloride (GaCl_3_) compound observed in spite of the reaction between Ga_2_O_3_ and HCl that will lead to the formation of GaCl_3_ compound as shown in Equation 1. FTIR results also confirmed the formation of Ga_2_O_3_ compound, and no other bonding such as Ga-Cl bonding to represent the impurity is detected. From the obtained results, it can be concluded that almost perfect reaction was taken place to form Ga_2_O_3_ structures.

## Conclusions

The growth of Ga_2_O_3_ nanostructures has been studied using electrochemical process, and the results were compared with hydrothermal process. In hydrothermal process, Ga_2_O_3_ nanorods were found to be formed in a heated electrolyte and they laid down on the surface of Si substrate. In electrochemical process with low molarity of Ga_2_O_3_, Ga_2_O_3_ nanodot-like structures without any nanorod structure were grown on silicon substrate. However, at high molarity of Ga_2_O_3_, nanorods were found to be formed in the electrolyte and their densities increase with the decrease of pH level, resulting to the mixture of nanodot and nanorod structures on Si substrate. From these result, Ga_2_O_3_ molarity and pH of electrolyte play significant role in determining the morphologies of the grown Ga_2_O_3_ structures.

## Competing interests

The authors declare that they do not have any competing interests.

## Authors’ contributions

NMG designed and performed the experiments, participated in the data analysis, and prepared the manuscripts. MRM helped in the PL and FTIR measurement. KY participated in the manuscript preparation. AMH conceived the study, designed the experiments, participated in the data analysis, and prepared the manuscript. All the authors read and approved the final manuscript.
